# Deciphering stromal cell interactions in osteosarcoma highlights CDKN2A and MMP14 as novel diagnostic and therapeutic biomarkers

**DOI:** 10.1186/s40001-026-03902-2

**Published:** 2026-01-27

**Authors:** Xiwen Peng, Haobo Jiang, Yicheng Sun, Jinwen Ge, Zhenhua Luo

**Affiliations:** 1 College of Integrated Traditional Chinese and Western Medicine, Hunan University of Chinese Medicine, Changsha, 410208 China; 2 School of Chinese Medicine, Hunan University of Chinese Medicine, Changsha, 410007 China; 3 Research Institute, Hunan University of Chinese Medicine, Changsha, 410000 China; 4 Department of Second Spine, The First Affiliated Hospital of Hunan University of Chinese Medicine, Changsha, 410000 China

**Keywords:** Osteosarcoma, Stromal cells, HdWGCNA, Molecular docking, Transcription factors, MiRNA

## Abstract

**Background:**

Osteosarcoma (OS) progression is critically influenced by the stromal microenvironment, yet we face a lack of reliable biomarkers. This study integrated single-cell and transcriptomic analyses to decipher stromal cell networks and identify novel diagnostic markers and therapeutic targets for OS.

**Methods:**

Using scRNA-Seq data and transcriptomic datasets obtained from the Gene Expression Omnibus (GEO), clustering analysis and communication analysis were performed with Seurat and CellChat packages, respectively. High-dimensional WGCNA (hdWGCNA) was applied to stromal cells to identify key gene modules. Hub genes from these modules were intersected with differentially expressed genes (DEGs) from DESeq2 analysis to pinpoint potential biomarkers. Their regulatory networks were predicted using the hTFtarget and ENCORI databases. Potential targeted drugs were screened via the DSigDB database and validated by molecular docking. Finally, functional assays were conducted using OS cell lines.

**Results:**

Single-cell transcriptomics analysis identified seven major cell subpopulations (macrophages, stromal cells, T cells, plasma cells, endothelial cells, plasmacytoid dendritic cells, and mast cells). Cell communication analysis showed that stromal cells and macrophages can interact via CD99–CD99. hdWGCNA analysis clustered 19 gene modules in stromal cells, among which modules M14, M15, and M17 were closely associated with OS and enriched in pathways, such as ossification, extracellular matrix organization, and skeletal system development. Two potential biomarkers (*CDKN2A* and *MMP14*) were screened. Transcription factor (TF) and miRNA regulatory network predictions indicated that both the two potential biomarkers were situated in a complex post-transcriptional regulatory network. Drug prediction and molecular docking results revealed that *MMP14* can stably bind to resveratrol. The proliferation, invasion, and migration capabilities of *MMP14*-silenced OS cell lines were significantly downregulated.

**Conclusion:**

This study identified *CDKN2A* and *MMP14* as potential OS biomarkers and elucidated their role within the stromal cell network, suggesting resveratrol as a candidate therapeutic molecule targeting *MMP14.* The present discoveries provided new insights for understanding the progression mechanisms and developing precise diagnosis and treatment strategies for OS.

## Introduction

Osteosarcoma (OS) is a highly aggressive malignant bone tumor that predominantly affects adolescents and children, with a global annual incidence of about 4.8 cases per million people [1, 2]. According to the WHO classification of soft tissue and bone tumors, the detection of even minimal neoplastic bone tissue is sufficient for OS diagnosis [3–5]. The primary management for OS involves wide surgical resection combined with chemotherapy. Radiation therapy is reserved for cases where tumors are unresectable. Although targeted therapies (e.g., VEGF receptor-tyrosine kinase inhibitors) have shown promising clinical efficacy, the 5-year overall survival rate remains modest at 66.2% [6–9]. Relapse and/or metastasis rates exceed 30%, and the 5-year survival drops below 25% for these patients due to chemoresistance or radioresistance [10]. The genetic heterogeneity and dynamic immunogenic characteristics of OS significantly affect therapeutic outcomes. For instance, although Immune Checkpoint Inhibitors have revolutionized the immunotherapy of solid tumors [11], anti-PD-L1 therapy exhibits only limited efficacy in OS [12]. Thus, there is an urgent need to elucidate the molecular mechanisms that drive OS progression and identify effective therapeutic targets to improve patient survival.

Advances in single-cell sequencing and multi-omics analyses have underscored that tumor progression is driven not only by genetic alterations within cancer cells, but also by dynamic interactions within the tumor microenvironment (TME) [13–15]. OS is a tumor characterized by its high heterogeneity and absence of well-defined driver mutations [16]. Stromal cells are increasingly recognized as central regulators of disease pathogenesis [17]. As essential components of the bone niche, stromal cells contribute to key processes, such as extracellular matrix remodeling, ossification, and local immune modulation, thereby creating a permissive milieu for tumor growth, invasion, and metastasis [18, 19]. For instance, crosstalk between OS cells and stromal cells can promote an acidic microenvironment, enhancing the expression of pro-inflammatory factors, such as IL-6 and NF-κB, which in turn accelerate invasive and metastatic behaviors [20]. Moreover, bidirectional signaling via extracellular vesicles and microRNAs further shows how stromal cells actively support OS progression [21, 22]. However, most studies focused on the phenotypic effects of stromal cells rather than systematically decoding their underlying molecular networks. A thorough dissection of stromal cell‑derived genes and regulatory pathways is therefore essential not only to uncover novel mechanisms of disease progression, but also to identify robust biomarkers that could guide early detection and targeted therapeutic strategies.

This study employed an innovative approach by integrating single-cell transcriptomics and bulk transcriptomic data to systematically decode the stromal cell networks within the microenvironment of OS, aiming to identify novel biomarkers and therapeutic targets. We first characterized the cellular landscape of OS, and then focused on analyzing stromal cell-associated gene modules and communication pathways. Key candidate biomarkers were subsequently validated through functional assays, and their regulatory networks and potential targeted drugs were explored. This work held significant value as it provided new mechanistic insights into OS progression, offered potential diagnostic markers for early detection and future therapeutic strategies, thereby contributing to the advancement of precision oncology for OS.

## Materials and methods

### Data acquisition

The OS single-cell dataset GSE162454 (with six tumor samples) were downloaded from GEO (Gene Expression Omnibus, https://www.ncbi.nlm.nih.gov/geo/) [23]. The OS-related dataset GSE99671 containing 18 OS tumor samples and 18 matched control samples was also downloaded from GEO database.

### scRNA-Seq analysis

In this study, the Seurat package [24] was used to read the scRNA-Seq data. After eliminating cells with fewer than 200 or more than 6,000 expressed genes, a total of 31,737 cells remained. Data standardization was performed using the SCTransform method, followed by PCA dimensionality reduction and batch effect removal using the harmony package. A KNN graph was developed based on the top 50 Prinicipal Components following UMAP dimensionality reduction, using the FindNeighbors function with Euclidean distance. Finally, the FindCluster function was applied for cell clustering at the resolution of 0.05. Cell types were determined based on the expression of known marker genes in the CellMarker database.

### Differential expression analysis between cell subpopulations

FindAllMarkers was used to identify genes specifically high-expressed in different cell subpopulations in OS, with the parameters set at logfc.threshold = 0.25, min.pct = 0.30, and only.pos = T.

### Cell communication analysis

To decipher intercellular communication involving stromal cells, we applied CellChat to identify significant ligand–receptor pairs between cell subpopulations and stromal cells in OS. The interaction type was selected as “Cell–Cell Contact.”

### High-dimensional WGCNA (hdWGCNA)

The hdWGCNA package was used to analyze single-cell transcriptomic data of OS. Genes expressed in at least 5% of cells were selected to create WGCNA objects. The KNN algorithm was employed to construct metacells, with a maximum of 10 cells shared by two metacells. Co-expression analysis was performed on the stromal cells, and the optimal soft threshold was calculated to construct the co-expression network. Genes exhibiting a high connectivity within the module were screened as hub genes.

### Functional enrichment analysis

Gene set was subjected to functional enrichment analysis using the KOBAS-i database (http://bioinfo.org/kobas) for identifying significant enriched Gene Ontology (GO) terms and Kyoto Encyclopedia of Genes and Genomes (KEGG) pathways (*p* < 0.05).

### Screening DEGs in OS

OS dataset samples were divided into disease and control groups. The DESeq2 package was used to calculate the relative expression of genes between the two groups, and DEGs were screened under the criteria |log_2_FC|≥ 1 and adjusted *p*-value < 0.01.

### Screening biomarkers in OS and construction of their transcription factor (TF) networks

Potential biomarkers for OS were identified by taking the intersection between the hub genes derived from high-dimensional weighted gene co-expression network analysis (hdWGCNA) with DEGs. Subsequently, potential TFs for the biomarkers were predicted using the hTFtarget database (https://guolab.wchscu.cn/hTFtarget/#!/), and their targeting miRNA were predicted using the Encori database (https://rnasysu.com/encori/). In both cases, only interactions supported by 3 or more experimental validations were retained. The results were imported into Cytoscape 3.8.0 to construct a TF regulatory network.

### Prediction of targeted drugs for OS and molecular docking

Potential targeted drugs for the identified biomarkers were predicted utilizing the DSigDB database via the R package Enrichr. Receptor protein crystal structures were obtained from the Uniprot database (https://www.uniprot.org/). 3D structures of targeted drugs were downloaded from the PubChem website (https://pubchem.ncbi.nlm.nih.gov/) as ligands. Drugs that lacked 3D structure or whose reported mechanism involved gene overexpression/knockdown were excluded from further analysis; subsequent candidates were selected in descending order according to the p value. The pymol software [25] was employed to perform dehydration, hydrogenation, and removal of small molecules on the molecules. Ligand structures were energy‑minimized using ChemBioOffice. Molecular docking was performed with AutoDockTools, and docking poses were screened based on a calculated binding energy threshold of < − 5 kcal/mol.

### Cell culture and transfection

hFOB1.19, U-2 OS, and SJSA-1 cell lines were purchased from ATCC (USA). Cells were maintained in DMEM medium supplemented with 10% FBS and cultured in a humidified incubator at 37 °C with 5% CO₂. Gene interference cell lines (si-target gene and si-NC) were transfected using Lipofectamine 3000 reagent (Invitrogen, USA). The sequences of si-target gene were as follows: si-MMP14#1: AAGATCAAAAACCGGAAAAGAGG; si-MMP14#2: GAGGAACAATCCCCTTTAACTCC.

### qRT-PCR

Total cellular RNA was extracted using the Trizol method. cDNA synthesis was performed with the Qiagen One-Step RT-PCR Kit (Qiagen GmbH, Germany), followed by real-time quantitative PCR analysis. Amplification was performed using the SYBR Green system on an ABI 7500 PCR instrument (Thermo Fisher Scientific, USA). The primers were as follows:

MMP14 (Forward: CCTTGGACTGTCAGGAATGAGG; Reverse: TTCTCCGTGTCCATCCACTGGT);

CDKN2A (Forward: CTCGTGCTGATGCTACTGAGGA; Reverse: GGTCGGCGCAGTTGGGCTCC);

GAPDH (Forward: GTCTCCTCTGACTTCAACAGCG; Reverse: ACCACCCTGTTGCTGTAGCCAA).

### Cell proliferation assay

Cell proliferation capacity was assessed using CCK-8 assay kit (Dojindo Laboratories, Japan). Cells were seeded into 96-well plates and added with 10 μL of reaction solution. Absorbance was measured at 450 nm to reflect cell viability.

### Cell invasion assay

The bottom of Transwell chambers (Corning, USA) was pre-coated with 30 μL of Matrigel. Cell suspensions were added to the upper chamber, while the lower chamber medium with FBS. After incubation at 37 °C for 48 h, non-transfected cells were removed. After using 4% paraformaldehyde to fix the cells, crystal violet was used to color the cells for 10 min. Finally, an inverted microscope was employed to observe and count invading cells.

### Scratch assay

Cells were planted into 6-well plates until confluent. Next, linear scratches were created using a pipette tip. After washing the cells twice with PBS to remove debris, serum-free medium was replaced to continue the culture. The cells were photographed at 0 h and 48 h. The scratch healing rate was calculated as: Healing rate = (Initial scratch width − current scratch width)/initial scratch width × 100% [26].

### Statistical analysis

All statistical data were analyzed using the R language (version 4.2.0) and GraphPad Prism software (version 8.0.2). The Wilcoxon test was used to calculate the differences between two groups of continuous variables, and analysis of variance (ANOVA) was applied to compare the experimental data. *p* < 0.05 was considered statistically significant.

## Results

### Single-cell atlas of OS

**Fig. 1:**
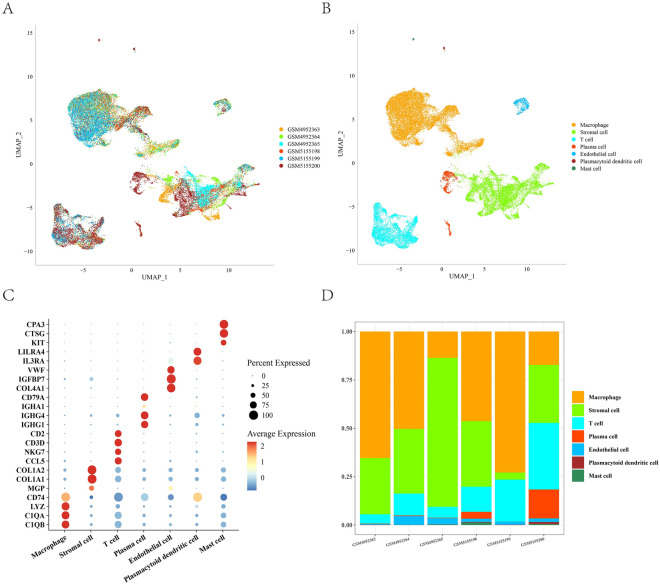
Single-cell atlas of osteosarcoma. **A** Distribution of osteosarcoma samples after batch removal. **B** UMAP plot of cell clusters in osteosarcoma. **C** Expression levels of marker genes in different cell subpopulations. **D** Proportions of cell subpopulations in each sample

This study identified seven major cell subpopulations in OS, including macrophages, stromal cells, T cells, plasma cells, endothelial cells, plasmacytoid dendritic cells, and mast cells (Fig. 1A, B). Cell identities were annotated based on established marker genes: macrophages (*C1QB*, *C1QA*, *LYZ*, and *CD74*); stromal cells (*MGP*, *COL1A1*, and *COL1A2*); T cells (*CCL5*, *NKG7*, *CD3D*, and *CD2*); plasma cells (*IGHG1*, *IGHG4*, *IGHA1*, and *CD79A*); endothelial cells (*COL4A1*, *IGFBP7*, and *VWF*); plasmacytoid dendritic cells (*IL3RA* and *LILRA4*); and mast cells (*KIT*, *CTSG*, and *CPA3*). A bubble plot visualizing marker expression across clusters and a bar chart depicting cell type proportions in the sample were plotted (Fig. 1C, D).

### Cell communication analysis

**Fig. 2:**
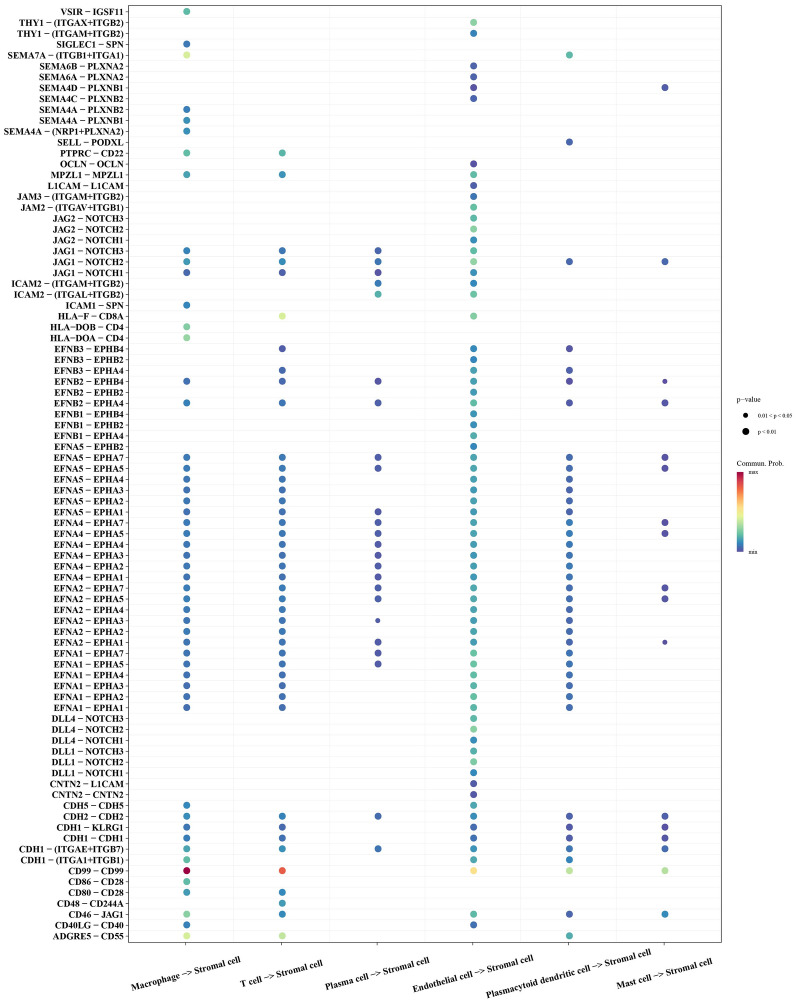
Receptor–ligand pairs regulating stromal cells in osteosarcoma

This study revealed extensive intercellular communication in OS through cell communication analysis. By extracting ligand–receptor information from each cluster, we identified a CD99-CD99-mediated interaction between stromal cells and macrophages (Fig. 2).

### hdWGCNA screening of gene modules associated with stromal cells in OS

**Fig. 3:**
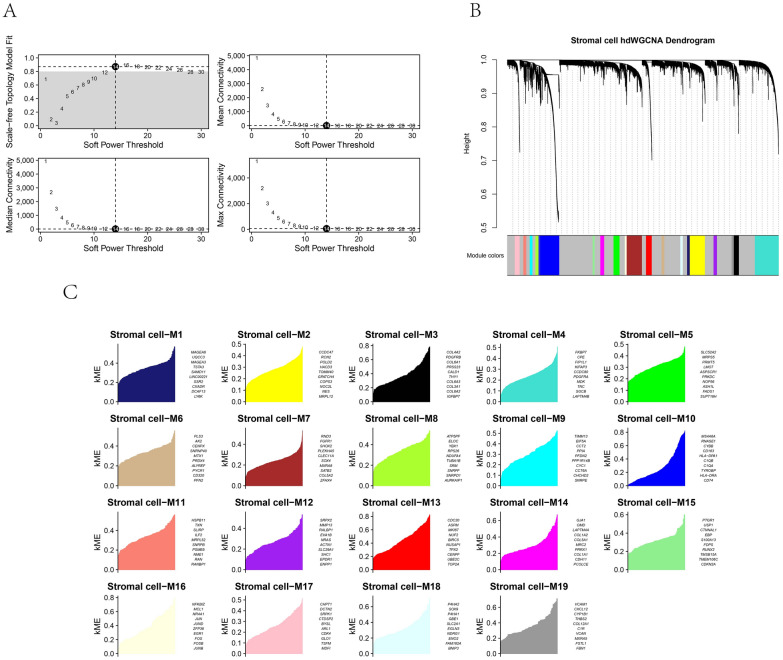
hdWGCNA analysis of gene modules in osteosarcoma. **A** Scaleless fitting index analysis for various soft threshold powers (β) and average connectivity analysis for various soft threshold powers. **B** Co-expression network diagram of gene clusters associated with each cell type. **C** Module partitioning, with the vertical axis representing kME values (connectivity of each gene based on feature genes) and the right side showing the hub genes of each module

**Fig. 4:**
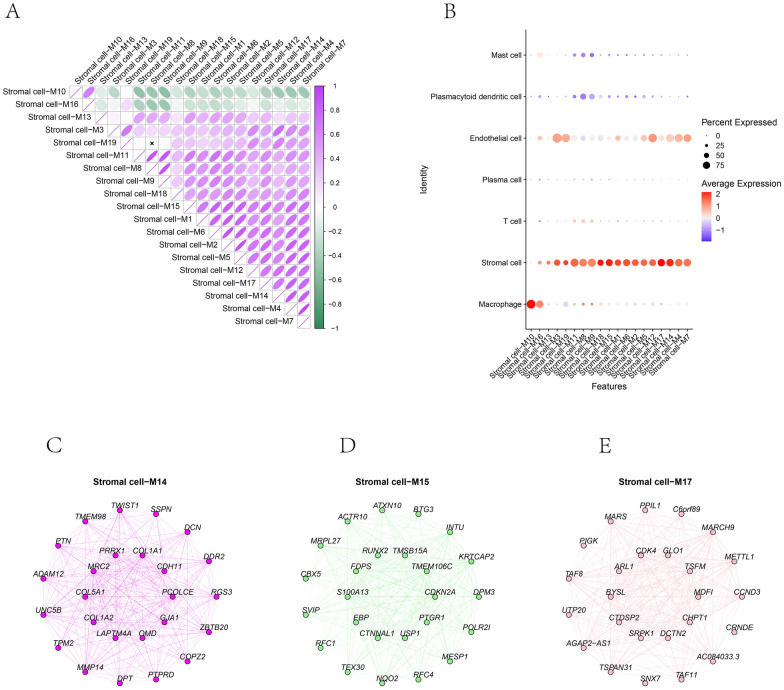
Expression of key modules in stromal cells. **A** Expression of genes within the module in different cells. **B** Module correlation matrix. **C–E** Co-expression network of hub genes within the module, with key genes in the inner circle and secondary key genes in the outer circle

**Fig. 5:**
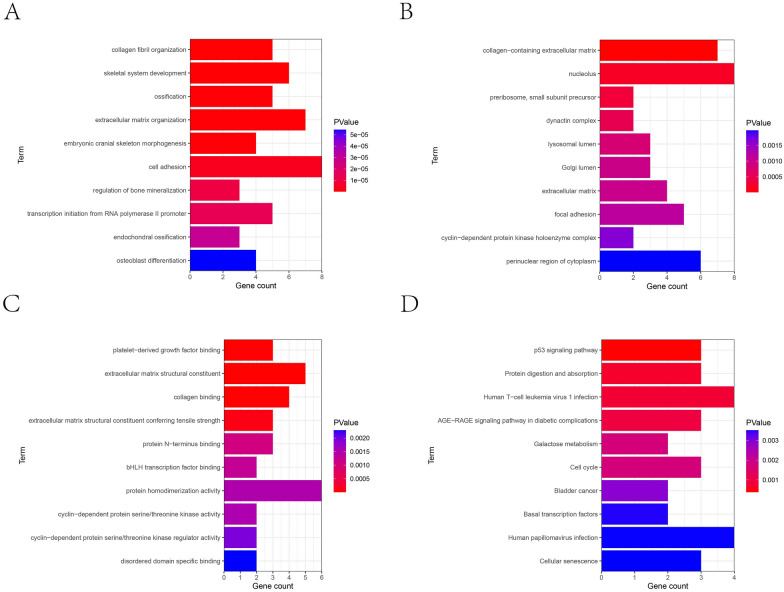
Enrichment analysis of stromal cell-related genes in osteosarcoma. **A** Gene GO enrichment analysis BP bar chart, with the x-axis representing the number of genes contained in the entry and the color representing the significance p value, increasing from blue to red. **B** Gene GO enrichment analysis CC bar chart. **C** Gene GO enrichment analysis MF bar chart. **D** Gene KEGG enrichment analysis bar chart

The optimal soft threshold of 14 was determined to construct the tool expression network on stromal cells (Fig. 3A, B). By calculating the module's eigenvalue (ME) and module connectivity, hub genes within the modules were screened and divided into 19 modules (Fig. 3C). Analysis of gene expression across cell types revealed that genes in modules M14, M15, and M17 had higher expression proportions and expression specific in stromal cells (Fig. 4A, B). Therefore, we focused on these three modules as key stromal-associated modules and constructed their intramodular hub gene networks (Fig. 4C–E). Enrichment analysis showed that the stromal cell-related key genes were significantly enriched in pathways such as ossification, extracellular matrix organization, and skeletal system development (Fig. 5A–D).

### Screening of DEGs associated with OS progression

**Fig. 6:**
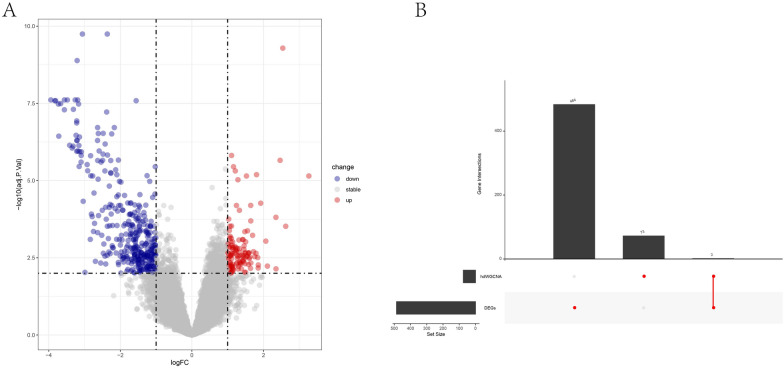
Differential expression analysis of osteosarcoma. **A** Volcano plot of differential analysis, with red representing differentially upregulated genes, blue representing differentially downregulated genes, and gray representing non-differentially expressed genes. **B** Upset plot of differentially expressed genes in osteosarcoma and hub genes of the hdWGCNA module

Differential expression analysis based on OS-related data ultimately identified 486 DEGs (127 upregulated genes and 359 downregulated genes) (Fig. 6A). In this study, the genes identified by hdWGCNA screening were intersected with the DEGs, yielding two significantly expressed stromal cell-related genes (*CDKN2A* and *MMP14*), which were used as potential biomarkers for OS (Fig. 6B).

### Construction of a TF regulatory network for potential OS biomarkers

**Fig. 7:**
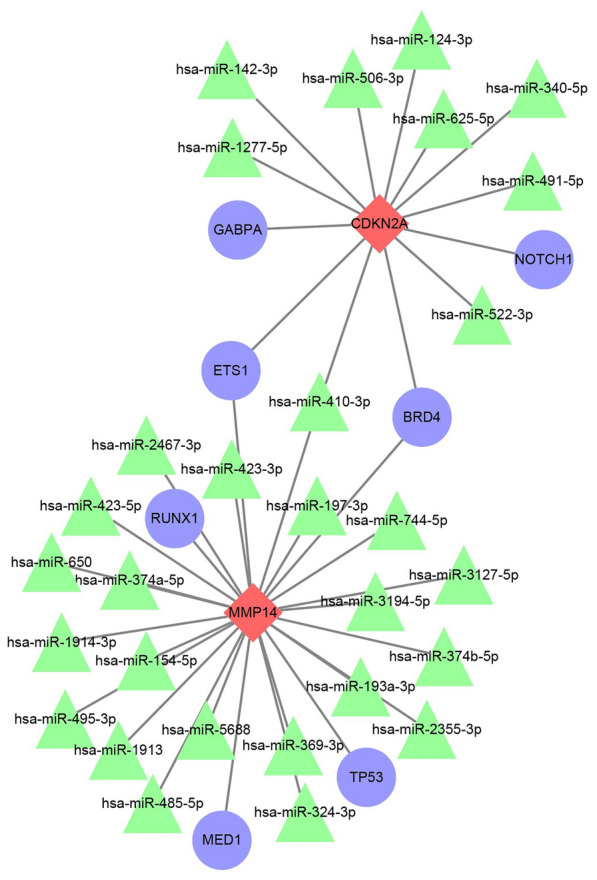
Transcriptional factor regulatory network of potential biomarkers for osteosarcoma. Red diamonds represent biomarkers, blue circles represent transcription factors, and green triangles represent miRNAs

TF prediction results showed that *CDKN2A* has four TFs, including *GABPA, NOTCH1, ETS1*, and *BRD4*, while *MMP14* had three corresponding TFs, including *RUNX1, TP53,* and *MED1*. *MMP14* had 21 target miRNAs validated by three or more experiments, while *CDKN2A* had nine. These regulatory interactions were integrated to Cytoscape to construct a TF regulatory network (Fig. 7).

### Prediction of potential therapeutic drugs for OS and molecular docking

**Fig. 8:**
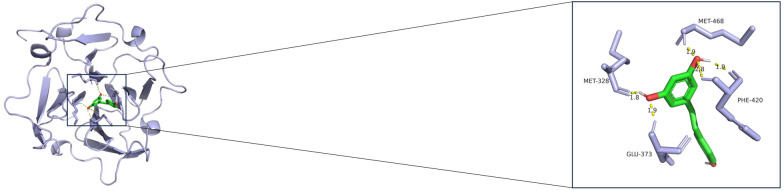
Molecular docking simulation of potential biomarkers for osteosarcoma. Molecular docking results for MMP14 and resveratrol. The blue molecule in the figure is the receptor protein, the green molecule is the drug small molecule, and the cyan green represents amino acids. The hydrogen bonds between the receptor protein and the drug small molecule are indicated by yellow dashed lines, with the numbers above representing the hydrogen-bond length in angstroms (Å)

Potential therapeutic drugs targeting the biomarkers for OS were predicted via the DSigDB database, and resveratrol was selected as the drug molecule for molecular docking. The results demonstrated a stable binding between *MMP14* and resveratrol, and the hydrogen-bond lengths were all within the normal range (Fig. 8).

### Regulatory role of the biomarkers at the cancer cell level in OS

**Fig. 9:**
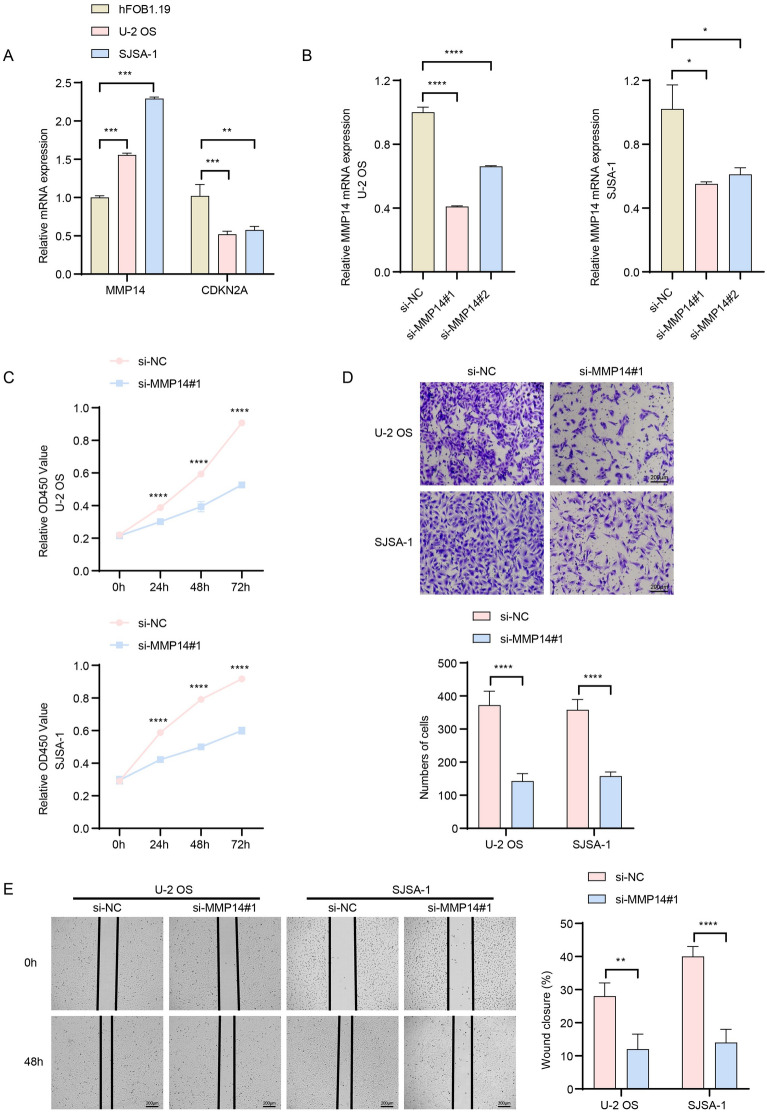
Regulatory role of biomarker MMP14 in the malignant phenotype of Osteosarcoma cell lines. **A** Expression of MMP14 and CDKN2A in cell lines U-2OS and SJSA-1 compared to hFOB1.19. **B** Construction of MMP14-silenced U-2OS and SJSA-1 cell lines. **C** CCK-8 assay measuring cell proliferation in U-2OS/si-MMP14 and SJSA-1/si-MMP14 cells. **D** Transwell assay assessing relative invasion levels in U-2OS/si-MMP14 and SJSA-1/si-MMP14 cells. **E** Scratch healing assay for relative migration levels in U-2OS/si-MMP14 and SJSA-1/si-MMP14. *p < 0.05, **p < 0.01, ***p < 0.001, ****p < 0.0001

This study measured the relative expression of multiple biomarkers in OS cell lines, revealing significant overexpression of *MMP14* in the cancer cells (Fig. 9A). We established cell lines U-2OS/si-*MMP14* and SJSA-1/si-*MMP14* to achieve MMP14 gene silencing (Fig. 9B). CCK-8 assay demonstrated that *MMP14* silencing significantly inhibited OS cell proliferation (Fig. 9C). Transwell and scratch healing assays revealed that MMP14 silencing markedly suppressed the migration and invasion capabilities of OS cell lines (Fig. 9D, E).

## Discussion

OS, a highly invasive bone malignancy common in adolescents, continues to show a poor prognosis despite multimodal therapy, underscoring the need to discover reliable biomarkers [27]. In recent years, the application of single-cell sequencing technology has provided unprecedented resolution for deciphering the TME of OS. For instance, by integrating single-cell transcriptomic and whole-exome sequencing data, Zhou et al. systematically characterized the cellular landscape of OS and identified a cancer-associated fibroblast (CAF) subpopulation linked to poor prognosis. This CAF subset was marked by high expression of genes involved in angiogenesis and ECM remodeling [12]. Meanwhile, regarding the tumor-immune microenvironment, researchers have also characterized the dynamic evolution of myeloid cells in primary and metastatic OS using single-cell technology, and identified a tumor-associated macrophage (TAM) subpopulation with a unique M2-like and pro-metastatic phenotype, which further emphasized the importance of immune-stromal crosstalk in the TME [28, 29]. Collectively, these studies highlighted the powerful capability of single-cell technology in unraveling cellular heterogeneity and intercellular interaction networks within the microenvironment of OS, laying a solid theoretical foundation and methodological validation for the rationale of the present study. Based on this, this study integrated single-cell and transcriptomic data to elucidate the cellular composition of OS and the key role of stromal cells. We identified *CDKN2A* and *MMP14* as key stromal cell-derived biomarkers in OS, situating them within specific TF and miRNA regulatory networks. Molecular docking predicted resveratrol as a potential *MMP14*-targeting agent, and functional validation confirmed that *MMP14* knockdown suppressed malignant phenotypes in OS cells. These findings provided new insights into stromal cell-driven mechanisms and offered potential targets for the precision diagnosis and therapy in OS.

The two biomarkers (*CDKN2A* and *MMP14*) identified by this study were not only closely linked to OS progression but were also functionally embedded within stromal cell-driven mechanisms. *CDKN2A*, a cyclin-dependent kinase inhibitor, played a critical role in stromal cell-mediated metabolic reprogramming. Its expression influenced lipid metabolism and energy homeostasis, which in turn modulated the stromal support for tumor growth and inflammatory signaling in the TME [30, 31]. Loss or mutation of *CDKN2A* enhanced stromal adaptability under metabolic stress, promoting a tumor-favorable niche that facilitated OS progression through mechanisms, such as altered glycolysis and macrophage M2 polarization [32, 33]. Similarly, *MMP14*, a transmembrane matrix metalloproteinase, was integral to stromal control over ECM remodeling and intercellular communication [34]. In OS-associated stromal cells, elevated *MMP14* activity contributed to ECM degradation, collagen breakdown, and the release of pro-invasive factors, thereby enabling tumor cell dissemination [35]. Furthermore, *MMP14* can activate signaling cascades, such as the Notch3 pathway, enhancing cancer stemness and therapeutic resistance within the stromal–tumor interface [36]. In OS, stromal-derived MMP14 also promoted angiogenesis and immune evasion by processing cytokines and chemokines, embedding it within a multi-dimensional network that supported the metastatic phenotype [37, 38]. Collectively, *CDKN2A* and *MMP14* could exemplify how stromal cells exerted pleiotropic effects on OS through metabolic reprogramming and matrix remodeling, highlighting these molecules as linchpins of microenvironment-driven pathogenesis and promising targets for stroma-based therapeutic intervention.

Additionally, this study further revealed that *CDKN2A* and *MMP14* were involved in a complex multi-level regulatory network through TF and miRNA prediction. TF analysis showed that *CDKN2A* was regulated by *GABPA*, *NOTCH1*, *ETS1*, and *BRD4*, all of which played key roles in cell proliferation, apoptosis, and signal transduction. For example, *GABPA* influenced cancer cell proliferation, migration, and invasion by regulating ferroptosis [39]. *NOTCH1* was also involved in pathways closely associated with distant metastasis in cancer cells [40]. *ETS1* drove the EGF-related pathway to induce glycolysis in epithelial cells, thereby affecting cancer cell migration and EMT [41]. These results also suggested that *CDKN2A* may be finely regulated through multiple signaling pathways in OS. *MMP14* was associated *RUNX1*, *TP53*, and *MED1*. Given that *TP53* was a classic tumor suppressor, while *RUNX1* and *MED1* played prominent roles in tumor invasion and transcriptional co-activation. Our findings suggested that *MMP14* may promote OS progression by interacting with tumor suppression and transcriptional activation networks [42–44]. Furthermore, by mining miRNA-target databases with stringent validation thresholds, we identified robust regulatory sites for both *MMP14* and *CDKN2A*, underscoring that these biomarkers were under extensive post-transcriptional control. In summary, the regulatory network constructed in this study demonstrated the synergistic role of TFs and miRNAs in the molecular mechanisms of OS, providing new insights into understanding the complexity of gene regulation and identifying potential intervention targets.

Molecular docking analysis was performed to explore potential interactions between the identified biomarker *MMP14* and bioactive compounds. The results indicated that resveratrol, a naturally occurring polyphenol with documented antioxidant, anti-inflammatory, and antitumor properties [45–47], could form stable hydrogen-bond interactions with *MMP14* within a reasonable binding distance. While this in silico prediction suggested a possible binding affinity, it should be noted that docking analysis alone cannot confirm target specificity or functional relevance in a biological context. Given the established role of *MMP14* in promoting invasion, metastasis, and EMT in OS [37, 48], further experimental validation is still needed to determine whether resveratrol exerted its antitumor effects, in part, through modulation of *MMP14* activity or related stromal signaling pathways. In addition, this study had several other limitations. For example, the analysis relied primarily on a single set of transcriptomic and single-cell data from public databases, with relatively homogeneous sample sources. The lack of validation across multiple platforms or independent cohorts may limit the generalizability of our findings. Second, although key biomarkers and their regulatory networks were preliminarily validated by cell experiments, their functions and pathological relevance still require further confirmation in animal models and clinical samples. Finally, as we focused on the stromal cell network without systematically integrating other microenvironment components, the study may fail to fully elucidate the entire landscape of microenvironment interactions. To address these limitations, future studies are encouraged to construct validation cohorts by integrating multicenter, multi-omics data, and conduct in vivo functional studies using gene-editing animal models. Simultaneously, by combining techniques such as spatial transcriptomics or multiplex immunofluorescence, spatial interaction relationships between stromal cells and components can be analyzed in situ, thereby elucidating the regulatory mechanisms and therapeutic targets of the microenvironment of OS in a more systematic and clinically relevant manner.

## Conclusion

In conclusion, this study leveraged single-cell transcriptomics to delineate the stromal cell landscape within the microenvironment of OS. Through integrated analysis, we identified *CDKN2A* and *MMP14* as key hub genes derived from stromal cell co-expression networks. In vitro functional validation confirmed the critical role of *MMP14* in promoting OS cell proliferation, invasion, and migration. Furthermore, we constructed the transcriptional and post-transcriptional regulatory networks for these biomarkers and proposed resveratrol as a candidate therapeutic agent targeting *MMP14* molecular docking. These findings provided new mechanistic insights into stroma-mediated OS pathogenesis and offered potential targets for advancing diagnostic and therapeutic strategies.

## Data Availability

The datasets generated and/or analyzed during the current study are available in the [GSE99671] repository, [https://www.ncbi.nlm.nih.gov/geo/query/acc.cgi?acc=GSE99671] and [GSE162454] repository, [https://www.ncbi.nlm.nih.gov/geo/query/acc.cgi?acc=GSE162454].

## Abbreviations

GEO: Gene expression omnibus
scRNA-Seq: Single-cell RNA sequencing
hdWGCNA: High-dimensional weighted gene co-expression network analysis
DEGs: Differential expressed genes
GO: Gene ontology
KEGG: Kyoto encyclopedia of genes and genomes
PCA: Principal component analysis
KNN: K-nearest neighbor
